# Supplementary material for: Juvenile reinstatement of TCF4 in Pitt-Hopkins syndrome model mice reveals a critical window for genetic intervention
Source: bioRxiv. 2025 Dec 24:2025.12.23.696277. Preprint. [Version 1] doi: 10.64898/2025.12.23.696277 (PMC12776099; doi:10.64898/2025.12.23.696277)
Supplement: Supplement 1 [file NIHPP2025.12.23.696277v1-supplement-1.pdf]

bioRxiv preprint doi: https://doi.org/10.64898/2025.12.23.696277; this version posted December 24, 2025. The copyright holder for this preprint (which was not certified by peer review) is the author/funder, who has granted bioRxiv a license to display the preprint in perpetuity. It is made available under aCC-BY 4.0 International license.

SUPPLEMENTARY INFORMATION

Supplementary Table 1. Primers used throughout study.

Genotyping *Tcf4*-LSL mice	F	CACATGAAGCAGCACGACTT
	R	AGTTCACCTTGATGCCGTTC
Mal	F	CTGGCCACCATCTCAATGT
	R	TGGACCACGTAGATCAGAGT
Tcerg1l	F	TGCTGCTAGCCAAAGAAGAA
	R	TCTGAACAAGACGAAACCTCTG
Tcf4	F	GCGAATCACATGGGACAGAT
	R	CAGCTGTTAAGGAAGTGGTCTC
Eif4a2	F	TCTCAATACAAGGCGCAAGG
	R	CTCTTTCCTTCTGGTCCATGTC
Glra3	F	TGCAAGAATCAGACCCAACT
	R	CAATGGAGCCAAAGCTGTTTAT
